# A Machine Learning and Cross-Validation Approach for the Discrimination of Vegetation Physiognomic Types Using Satellite Based Multispectral and Multitemporal Data

**DOI:** 10.1155/2017/9806479

**Published:** 2017-06-11

**Authors:** Ram C. Sharma, Keitarou Hara, Hidetake Hirayama

**Affiliations:** ^1^Department of Informatics, Tokyo University of Information Sciences, 4-1 Onaridai, Wakaba-ku, Chiba 265-8501, Japan; ^2^Graduate School of Informatics, Tokyo University of Information Sciences, 4-1 Onaridai, Wakaba-ku, Chiba 265-8501, Japan

## Abstract

This paper presents the performance and evaluation of a number of machine learning classifiers for the discrimination between the vegetation physiognomic classes using the satellite based time-series of the surface reflectance data. Discrimination of six vegetation physiognomic classes, Evergreen Coniferous Forest, Evergreen Broadleaf Forest, Deciduous Coniferous Forest, Deciduous Broadleaf Forest, Shrubs, and Herbs, was dealt with in the research. Rich-feature data were prepared from time-series of the satellite data for the discrimination and cross-validation of the vegetation physiognomic types using machine learning approach. A set of machine learning experiments comprised of a number of supervised classifiers with different model parameters was conducted to assess how the discrimination of vegetation physiognomic classes varies with classifiers, input features, and ground truth data size. The performance of each experiment was evaluated by using the 10-fold cross-validation method. Experiment using the Random Forests classifier provided highest overall accuracy (0.81) and kappa coefficient (0.78). However, accuracy metrics did not vary much with experiments. Accuracy metrics were found to be very sensitive to input features and size of ground truth data. The results obtained in the research are expected to be useful for improving the vegetation physiognomic mapping in Japan.

## 1. Introduction

Vegetation has been classified according to a number of criteria, such as climate [1], physiognomy [2], dominant species [3], combination of climate pattern and physiognomy [4], and physiognomic-floristic hierarchy [5]. Physiognomy means overall structure, physical appearance, and growth forms (herbs, shrubs, and trees) of the vegetation. It is descriptive of the size, leaf traits (needle-shaped or broadleaved), and phenology (deciduous or evergreen) of the dominant species [6]. Vegetation has been threatened by shifting of its zones and floristic decompositions under the influence of climate change [7–12]. Therefore, discrimination of the vegetation physiognomic characteristics is relevant to tracking the changes in vegetation structure and composition, thus understanding the vegetation responses to changes in environmental conditions.

Different attempts have been made for the classification and mapping of vegetation by exploiting the remote sensing data. Major sources of the remote sensing data are the imageries obtained from satellites or aircrafts. Both the multispectral and hyperspectral satellite data have been used [13–17]. More recently, vegetation mapping by using near surface multispectral, hyperspectral, or lidar imaging from manned or unmanned aircrafts is growing [18–20]. Radar imagery from the satellites is another viable data source for the vegetation mapping [21–24]. The discrimination and classification of the vegetation using the remotely sensed imagery involve with multiple image processing and classification techniques. Though some researchers have reported satisfactory results using multiple spectral mixture analysis [25], digital image enhancements [26], temporal image fusion [27, 28], and texture based classifications [29], supervised classification is probably the mostly used method for the vegetation classification. A number of supervised classifiers such as maximum likelihood method [30], decision trees [31], Support Vector Machines [32], fuzzy learning [33], Random Forests [34, 35], and Neural Networks [36–38] have provided promising results in different regions. However, most of these studies have not dealt with the discrimination and validation of all kinds of vegetation physiognomic classes such as Evergreen Coniferous Forest, Evergreen Broadleaf Forest, Deciduous Coniferous Forest, Deciduous Broadleaf Forest, Shrubs, and Herbs in a study area. The performance of existing land cover maps is limited for the discrimination of vegetation physiognomic types [39].

The discrimination of vegetation physiognomic types from remotely sensed data, though immensely important for detecting changes in vegetation structure and composition, is challenging. The Moderate Resolution Imaging Spectroradiometer (MODIS) on board the Terra and Aqua satellites provides a unique collection of time-series of the surface reflectance. This paper presents the performance and evaluation of a number of machine learning classifiers with respect to the time-series of the MODIS surface reflectance data for achieving an improved discrimination between the vegetation physiognomic types.

## 2. Materials and Methods

### 2.1. Preparation of Ground Truth Data

The existing geolocation data of the plant communities accessed from the Nature Conservation Bureau of the Ministry of Environment, Japan, were used for the preparation of ground truth data. These data were originally collected by field inspection of the plant communities according to the association of vegetation, the diagnostic/dominant species occurrence in the uppermost (and understory) stratum. We converted the plant community types into vegetation physiognomic types by studying the physiognomic characteristics of plant communities. The geolocation points were visually inspected with Google Earth based very-high-resolution time-lapse images available between 2012 and 2014, and the points representing a large homogenous (at least a single MODIS pixel size) area were finally selected. In this way, 300 geolocation points for each physiognomic class were prepared. This research deals with six vegetation physiognomic classes: Evergreen Coniferous Forest (ECF), Evergreen Broadleaf Forest (EBF), Deciduous Coniferous Forest (DCF), Deciduous Broadleaf Forest (DBF), Shrubs, and Herbs. The classification scheme adopted in the research is described in Table 1.

### 2.2. Processing of Satellite Data

Terra/Aqua satellite based MODIS Surface Reflectance 8-Day Level 3 Global 500 m data sets (MOD09A1 and MYD09A1) available over Japan in year 2013 were processed and used in the research. The MOD09A1 and MYD09A1 products provide an estimate of the surface spectral reflectance of bands 1–7 (Red, Near Infrared, Blue, Green, Mid Infrared, Shortwave Infrared 1, and Shortwave Infrared 2) as it would be measured at ground level in the absence of atmospheric scattering or absorption. Three spectral indices, Normalized Difference Vegetation Index (NDVI; [40]), Enhanced Vegetation Index (EVI; [41]), and Land Surface Water Index (LSWI; [42]), were also calculated for each scene. The 8-day data sets containing surface reflectance in seven bands and three spectral indices were composited using monthly and percentile based techniques. Multiple percentiles (0, 10, 20, 30, 40, 50, 60, 70, 80, 90, and 100) and monthly median composites (January to December) data were calculated pixel by pixel for each dataset. Altogether, 230 features (input layers) were prepared and deployed for the machine learning and cross-validation. The input features are described in Table 2.

### 2.3. Machine Learning and Cross-Validation

A set of experiments comprised of a number of supervised classifiers (*k*-Nearest Neighbors (KNN), Gaussian Naive Bayes (GNB), Random Forests (RF), Support Vector Machines (SVM), and Neural Network-Multilayer Perceptron (MLP)) with different model parameters was assessed for better discrimination of vegetation physiognomic types. For in-depth description of the machine learning algorithms, the OpenCV (http://opencv.org/), an optimized C/C++ programming library for computer vision, machine learning, and robotics, is referred. Experiments conducted in the research are listed in Table 3.

First of all, the given features were divided into 10-fold of samples. For each fold of samples, the learning was carried out only for nine folds, whereas the remaining one fold was used for the validation. However, inside the cross-validation loop, the best-scoring features (training) were selected based on univariate statistical test. We used the Analysis Of Variance *F*-value between physiognomic classes and features (training) as the univariate statistical test. The features (training) were grouped into 1–230 set(s) of best-scoring features. For instance, the first set included a single highest scored feature, whereas the last set included all 230 features. For each set of important features, the machine learning model established with the training folds was used to predict the physiognomic classes with the validation fold. The least number of best-scoring features that provided the highest kappa coefficient was noted as the optimum number of features. The Standard Deviations of the overall accuracy and kappa coefficient across the 10-fold cross-validation loop in the case of optimum number of features were also calculated. Finally, the predictions were collected from cross-validation loop; and the validation metrics, confusion matrix, overall accuracy, and kappa coefficient, was calculated with the given physiognomic labels. The overall accuracy—sum of true positives and true negatives divided by number of validation points—measures correctness of the classification. Kappa coefficient measures interrater agreement by counting the proportion of instances that predictions agreed with the validation data (observed agreement) after adjusting for the proportion of agreements taking place by chance (expected agreement) [43]. The same processing was conducted for each experiment.

## 3. Results and Discussion

### 3.1. Cross-Validation Results

The performance of 10 experiments conducted in the research is summarized in Table 4. The accuracy metrics, overall accuracy and kappa coefficient, were calculated based on 10-fold cross-validation method. The Standard Deviations (SD) of the overall accuracies and kappa coefficients across the 10-fold cross-validation in the case of optimum number of features are also shown in Table 4. Experiment number 6 using the Random Forests classifier yielded highest overall accuracy (0.81) and kappa coefficient (0.78) with 160 input features. However, it should be noted that the overall accuracy metrics did not vary much with experiments. Experiments using KNN, GNN, RF, SVM, and MLP yielded 0.75, 0.64, 0.78, 0.76, and 0.76 highest kappa coefficients, respectively. The MLP-based experiments were highly sensitive to the standardization of the features compared to other experiments. Therefore, features were standardized by removing the mean and scaling to unit variance in the case of MLP-based experiments.

The optimum number of features, the set of lowest number of input features yielding highest kappa coefficient, differed widely from experiments. However the optimum number of features utilized by all experiments was very large. For instance, highest kappa coefficient (0.78) was obtained by using 160 input features out of 230 total features in the case of Experiment number 6. Therefore, time-series of the spectral features is important for discriminating the vegetation physiognomic classes. The selection of optimum features not only provides the best features required for discriminating the classes but also reduces the computation time and efforts [44].

### 3.2. Discrimination between Physiognomic Classes

**Figure 1:**
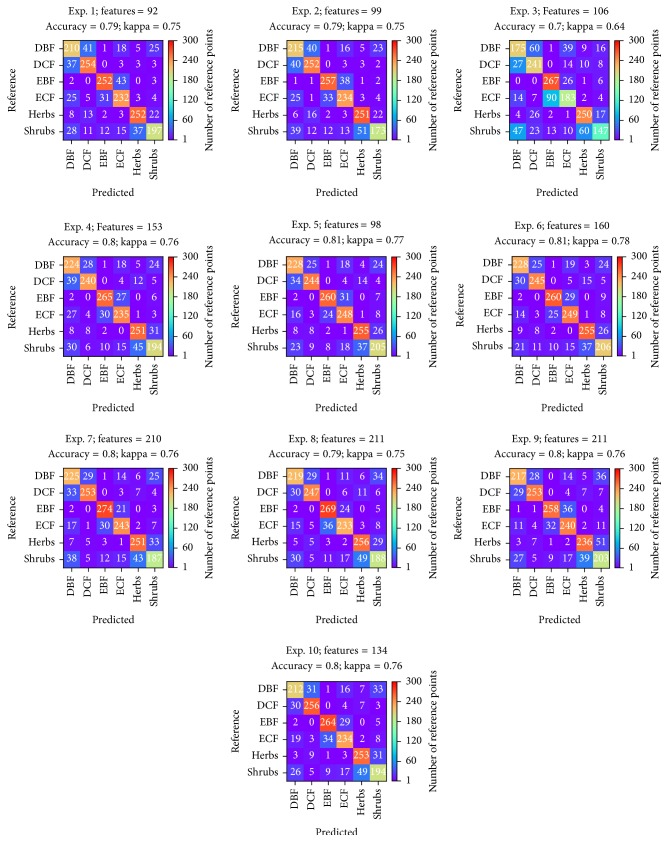
Confusion matrices for each experiment computed in the case of optimum number of features.

We computed the confusion matrices using the 10-fold cross-validation method. All experiments used the same size of ground truth data sets (300 for each physiognomic class). The ground truth data sets were well distributed all over the country. Six physiognomic classes evaluated under the research were Evergreen Coniferous Forest (ECF), Evergreen Broadleaf Forest (EBF), Deciduous Coniferous Forest (DCF), Deciduous Broadleaf Forest (DBF), Shrubs, and Herbs. The confusion matrices computed with the optimum number of features for each experiment are 163 plotted in Figure 1. The confusion matrices showed that none of the experiments could discriminate between DBF and DCF, between EBF and ECF, and between Herbs and Shrubs efficiently. Among 10 experiments conducted in the research, experiments using Random Forests, Support Vector Machines, and Neural Networks provided better discrimination between the challenging classes. It is still difficult to discriminate between the coniferous and broadleaved forests though the phenological discrimination between coniferous and broadleaved forests: DCF versus ECF or DBF versus EBF could be enhanced by utilizing time-series of the MODIS data.

### 3.3. Effect of Input Features

**Figure 2:**
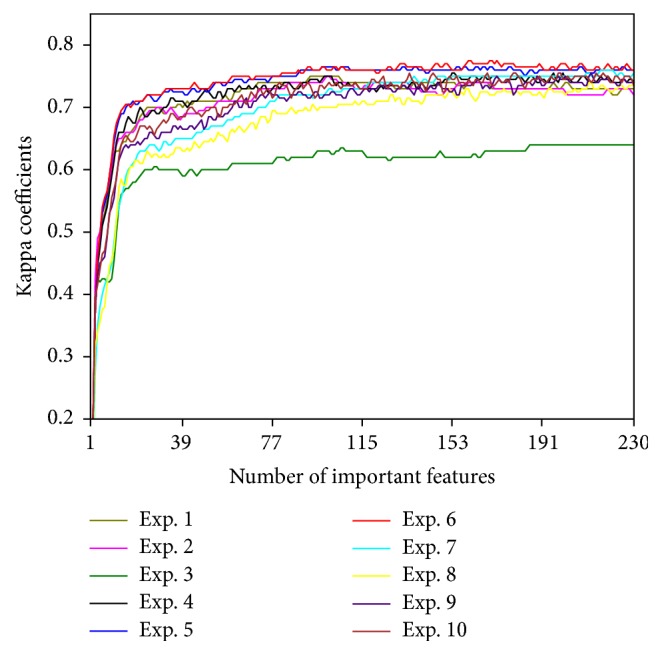
Variation of the kappa coefficient by increasing the number of input features.

The variation of the kappa coefficient by increasing the number of input features in the case of ground truth data sets with size 300 for each experiment is shown in Figure 2. Kappa coefficients increased by increasing the number of important features to some extent in all experiments, after which it saturated. Kappa coefficients were not highest by merely using all input features. Therefore, a combination of important features was found to be crucial for achieving the highest accuracy rather than just using the large number of features. Similar results were obtained in the case of ground truth data sets with sizes 200 and 100.

Large impact of feature selection on classification accuracy has also been reported in other land cover classification researches [44–48]. Since the countrywide discrimination of vegetation physiognomic types is challenging, selection of the important features should not be neglected.

### 3.4. Effect of the Ground Truth Data Size

**Figure 3:**
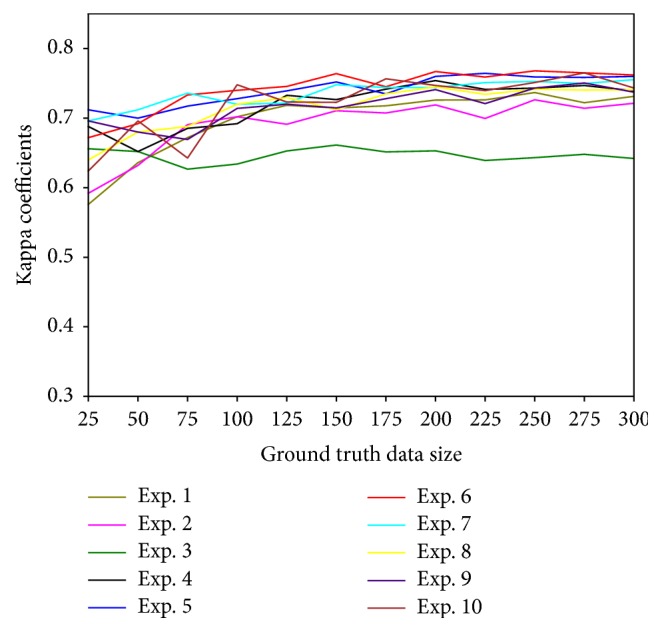
Variation of the kappa coefficient by increasing the ground truth data size.

The size of available ground truth data is usually limited as the collection of field data requires lots of time, efforts, and costs. The classifier providing highest accuracy metrics by using less size of the ground truth data would be preferred. To analyze effect of the ground truth data size on the accuracy, the ground truth data sets of size 300 available in the research for each physiognomic class were randomly sampled into 12 sets: 25, 50, 75, 100, 125, 150, 175, 200, 225, 250, 275, and 300. For each set, 10 experiments were conducted and the accuracy metrics were calculated using the 10-fold cross-validation method. The maximum kappa coefficients obtained from each experiment with respect to different data size are plotted in Figure 3.

As demonstrated in Figure 3, kappa coefficients are generally increased in all experiments by increasing the size of ground truth data. This analysis implies that large size of ground truth data is crucial to obtain higher accuracy. However, kappa coefficients did not increase in all experiments just by increasing the size of ground truth data. Therefore, optimized selection of the size of ground truth data with respect to the classifiers is important. The impact of the ground truth data size on the classification accuracy has also been discussed in other studies [49, 50].

### 3.5. Uncertainties and Limitations

Results obtained in the research may be prone to a number of uncertainties arising from ground truth data, satellite data, and computation efforts. Discrimination of vegetation physiognomic types using moderate resolution satellite data such as from the MODIS is affected by mixed pixel effect. The ground truth data sets used in the research were prepared from the large homogenous areas. Therefore, the cross-validation accuracies obtained in the research solely based on homogenous physiognomic classes may be lower in the field application. Utilization of high-resolution satellite data in future could minimize errors pertaining to homogeneity of the ground truth data sets and mixed pixel effects. Only highest quality surface reflectance data from MODIS was used by masking out the pixels affected by clouds, cloud shadows, cirrus, and large solar zenith angles using separate quality band descriptions available in the data sets. The seamless highest quality data may not be available throughout the country due to atmospheric effects. Comparison of the supervised classifiers as conduced in the research is not complete as only commonly used classifiers and model parameters were assessed. Comprehensive comparison of the supervised classifiers is certainly out of scope of the research. Nonetheless, evaluation results are consistent to other large studies. For example, Fernández-Delgado et al. 2014 [51] found Random Forests as the better classifier than Support Vector Machines or Neural Networks after rigorous comparison of 179 classifiers (machine learning algorithms) using 121 different data sets.

## 4. Conclusions

A set of machine learning experiments comprised of a number of supervised classifiers (*k*-Nearest Neighbors, Gaussian Naive Bayes, Random Forests, Support Vector Machines, and Multilayer Perceptron) with different model parameters was conducted to assess how the discrimination of vegetation physiognomic classes varies with classifiers, input features, and ground truth data size. The cross-validation method showed that the Random Forests provided highest overall accuracy (0.81) and kappa coefficient (0.78). However, accuracy metrics did not vary much with experiments. Optimum number of features, the set of lowest number of input features yielding highest kappa coefficient, were large (more than 92) in all experiments. The large number of optimum features required by the experiments implied that multitemporal satellite data are crucial for discriminating the vegetation physiognomic types. Kappa coefficients were not highest by merely using all input features. Therefore, combination of the important features was found to be crucial for achieving the highest accuracy rather than just using the large number of input features. Generally, the kappa coefficient increased in all experiments by increasing the size of ground truth data sets. Still, discrimination of some classes especially between the coniferous and broadleaved forests was not adequate which requires further exploration in future.

## Figures and Tables

**Table 1:** Vegetation physiognomy types used in the research.

Physiognomy types	Description
(1) Evergreen Coniferous Forest (ECF)	Forests dominated by conifer trees that retain leaves throughout the year
(2) Evergreen Broadleaf Forest (EBF)	Forests dominated by broadleaf trees that retain leaves throughout the year
(3) Deciduous Coniferous Forest (DCF)	Forests dominated by conifer trees that shed leaves seasonally
(4) Deciduous Broadleaf Forest (DBF)	Forests dominated by broadleaf trees that shed leaves seasonally
(5) Shrubs	Woody vegetation either evergreen or deciduous with less than 3 meters tall and more than 10% cover
(6) Herbs	Vegetation land covered by natural grasses or herbs with cover over 10%

**Table 2:** Description of the input features used in the research.

Features	Temporal composites		Subtotal features
	Monthly	Percentiles	
Spectral: Red, Near Infrared, Blue, Green, Mid Infrared, Shortwave Infrared 1, and Shortwave Infrared 2	12 × 7	11 × 7	161
Spectral indices: NDVI, EVI, and LSWI	12 × 3	11 × 3	69

Total features			230

**Table 3:** List of experiments conducted in the research.

Experiments	
1	*k*-Nearest Neighbors (neighbors = 5)
2	*k*-Nearest Neighbors (neighbors = 10)
3	Naive Bayes (algorithm = Gaussian)
4	Random Forests (trees = 10)
5	Random Forests (trees = 50)
6	Random Forests (trees = 100)
7	Support Vector Machines (kernel = linear)
8	Multilayer Perceptron (hidden units = 100; hidden layers = 1)
9	Multilayer Perceptron (hidden units = 100; hidden layers = 3)
10	Multilayer Perceptron (hidden units = 150; hidden layers = 5)

**Table 4:** Cross-validation results (size of ground truth data sets for each class = 300). The Standard Deviations (SD) across the 10-fold cross-validation in the case of optimum number of features are also shown.

Number of experiments	Max. overall accuracy	Overall accuracy SD	Max. kappa coefficient	Kappa coefficient SD	Optimum number of features
1	0.79	0.03	0.75	0.04	92
2	0.79	0.03	0.75	0.04	99
3	0.70	0.03	0.64	0.04	106
4	0.80	0.03	0.76	0.03	153
5	0.81	0.03	0.77	0.03	98
6	0.81	0.03	0.78	0.03	160
7	0.80	0.03	0.76	0.04	210
8	0.79	0.04	0.75	0.05	211
9	0.80	0.03	0.76	0.04	211
10	0.80	0.03	0.76	0.04	134
